# Sequential Application of Column Leaching and Plant Uptake Tests to Assess the Effect of Various Commercial Amendments on Cu Immobilization in Ultra-High Cu-Contaminated Soil

**DOI:** 10.3390/toxics10040185

**Published:** 2022-04-10

**Authors:** Tuan-Nguyen Quoc, Myung-Chae Jung

**Affiliations:** Environmental Geochemistry Laboratory, Department of Energy Resources and Geosystem Engineering, Sejong University, 209, Neungdong-ro, Gwangjin-gu, Seoul 05006, Korea; nqt18@sejong.ac.kr

**Keywords:** column leaching test, plant heavy metal uptake, soil amendments, Cu-contaminated soil, heavy metal immobilization

## Abstract

The presence of copper (Cu)-contaminated soil has increased recently due to agricultural and industrial activities. Immobilization techniques using soil amendments have attracted significant research because of their cost-effectiveness, eco-friendliness, and community acceptance. This study used various commercial amendments, including magnetite (M), talc (T), activated carbon (AC), and cornstarch (CS), to immobilize Cu in soil contaminated by acidic waste materials with Cu in Korea (9546 ± 5 mg/kg). To evaluate the immobilizing effect of these amendments, this study applied a sequential process of column leaching and plant uptake tests to observe the ability of Cu to remain in soil with and without amendments through the Cu removal rate. The amendments were characterized by SEM, XRD, and specific surface area and applied to the soil at a rate of 2% (*w/w*). The first stage of evaluation, i.e., the column leaching test, was conducted by continuously pumping distilled water (DW) for 28 days, and the second stage of evaluation, i.e., the plant uptake test, was started immediately after by planting 10-day-old lettuce seedlings for 28 days. The experimental results showed that all of the amendments had a significant effect on Cu immobilization Cu in soil (*p* < 0.05), and the T treatment showed the highest efficiency in Cu immobilization, with only 47.0% Cu loss compared to 73.5% in the control soil when assessed by sequential column leaching and plant uptake tests. In conclusion, this study provides an effective assessment method to evaluate the effect of amendments on Cu immobilization in soil, as well as providing feasible options to immobilize Cu using commercial amendments.

## 1. Introduction

Currently, the rapid growth of urbanization and industrialization has become a global concern for heavy metal contamination in soil [1,2,3]. Copper is one of the trace elements in soil. High levels of Cu concentration in the environment can affect environmental quality by reducing crop production, and groundwater toxicity can further cause serious human health risks [4,5,6]. Therefore, the remediation of heavy-metal-contaminated soil is an urgent priority to protect food production and water quality. 

In recent decades, many techniques have been proposed to remedy heavy-metal-contaminated soils [7,8]. One of the most cost-effective remediation strategies for heavy-metal-contaminated soils is metal immobilization using a wide range of chemical amendments [9,10]. Recently, commercial amendments such as magnetite, talc, activated carbon, and cornstarch have attracted attention for the immobilization of heavy metals in soil due to their ready availability, isomorphic substitution, permanent negative charges on the surface, environmental compatibility, feasibility, and promise [7,11,12,13,14,15,16,17]. For example, recent research reported that commercial magnetite significantly immobilizes As in soil, reducing As by mobility up to 92.3% in the toxicity characteristic leaching procedure test [18]. Talc is one of the clay minerals that has been applied in research as part of sepiolite, which can immobilize Cd in soil, reduce plant toxicity, and enhance the soil microbial community [19]. Additionally, activated carbon has also been reported due to its effect on immobilizing Hg and other heavy metals such as Ni, Zn, and Cu in contaminated soil [12,20]. In addition, it has been reported that cornstarch, known as a biopolymer, has a significant effect on reducing the mobility of Zn in contaminated soil, as well as enhancing the microbial community in soil [10]. 

Due to the mobility and bioavailability of heavy metals in soil, they can easily leach into groundwater or accumulate in plants. Therefore, the immobilization technique is necessary to reduce the mobility and bioavailability of heavy metals in soil, rather than the total amount [21,22]. To assess the effect of amendments on immobilizing heavy metals in soil, chemical extractants such as HCl, NH_4_OAc, CaCl_2_, and EDTA have often been applied [23,24]. On the other hand, soil washing and phytoextraction have been used to remove heavy metals from soil. Therefore, this study applied a sequential process of column leaching and plant uptake tests to evaluate the effect of various soil amendments, including magnetite, talc, activated carbon, and cornstarch, on Cu immobilization by considering the removal rate of Cu in the presence of amendments. 

## 2. Materials and Methods

### 2.1. Characterization of Soil and Amendments

Soil samples were collected at a depth of 0–20 cm from an area contaminated by acidic waste materials with Cu in Korea. This area was selected because of the high concentration of Cu in the soil and the possibility of Cu leaching into the environment, making it very promising for the application of soil amendments for Cu immobilization. Soil samples were stored in plastic bags and transferred to the laboratory for future experiments. In the laboratory, soil samples were air-dried and sieved to have particle size fractions <2 mm. The pH values of soil and amendments were determined using a pH meter at a 5:1 water/soil ratio (*v*/*v*). Soil texture was analyzed using the sedimentation method [25] and classified by the United States Department of Agriculture (USDA). To analyze the total concentration of Cu in soil and amendments, samples (1.5 g) were digested in a solution of HNO_3_/HCl (3:1 *v*/*v*) at 70 °C and analyzed using atomic absorption spectrometry (AAS, AA240, Varian, Australia). Quality assurance of the sample analysis process was confirmed using certified reference materials (BAM 112a) for aqua regia extractable trace elements in soil with a Cu concentration of 75.5 ± 3.1 mg/kg. However, more appropriate certified reference materials with high concentrations of Cu and comparable soil properties were not available. 

Four kinds of commercial amendments, including magnetite (M), talc (T), activated carbon (AC), and cornstarch (CS), were ordered from Daejung Chemical Co. Ltd, Seoul, Korea. The specific surface area (SSA) of the amendments was measured by N_2_ adsorption using the Micromeritics BET method of Tristar 3000 (U.K.) [26]. The morphology of each amendment was examined using scanning electron microscopy (SEM). X-ray diffraction (XRD) was used to identify the crystalline phase of the amendments. 

### 2.2. Sequential Application of Column Leaching and Plant Uptake Tests

The first stage of this study was conducted in the column leaching test. Firstly, the four amendments, including M, T, AC, and CS, were mixed with Cu-contaminated soil at a rate of 2% *w*/*w* (amendment/soil) and incubated in the dark for two months to achieve equilibrium. After that, the prepared soil (with amendments) and control soil (without amendments) were weighed to 600 g and then placed in a column 5 cm in diameter and 30 cm in height. A plastic mesh (D = 0.2 mm) was placed at the bottom of the column to retain the soil. Washing solutions using distilled water (DW) were passed through the corresponding soil columns by using a peristaltic pump at a 1 mL/min flow rate. The effluent was collected and analyzed for pH and Cu content (mg/L) using AAS for each interval period. After 28 days consecutively, the remaining soil was transferred to the second stage (plant uptake test) by growing lettuce.

In the second stage, a pot experiment was carried out in the research greenhouse of Sejong University, located in Seoul, the Republic of Korea, to investigate the effect of soil amendments. The pots, measuring 12 cm × 8 cm × 10 cm (upper diameter × bottom diameter × height), were separately filled with the contaminated soils that were washed in the first stage. Two 10-day-old lettuce seedlings from unpolluted soils were transplanted into each experimental pot and watered 2 times a week (100 mL d^−1^) with DW. After 28 days of planting, plants were harvested, and soil was collected for further analysis. Harvested plants were separated as part of root and shoot samples. Plant samples were rinsed with tap water and then with DW. The samples were oven-dried (40 °C) for 7 days, ground, and stored in a plastic bag at room temperature before analysis. To measure the total content of Cu in plant tissues, samples were digested in a solution of 1 mL of H_2_O_2_ and 5 mL of HNO_3_ (70% *w*/*w*) on a hot plate at 120 °C for 1 h [27]. The resultant solutions were filtered using a cellulose membrane filter and then analyzed using AAS.

The removal rate of Cu was calculated by the formula:

(1) $$\mathrm{Romoval}\;\mathrm{rate}(\%)=(C_{0}-C_{i})/C_{0}$$

where C_0_ is the initial concentration of Cu, and C_i_ is the Cu concentration remaining in the soil when assessed by sequential column leaching and plant uptake tests.

### 2.3. Statistical Analysis

Soil and plant Cu concentration data were calculated from triple independently replicated experiments. Data were statistically analyzed by applying one-way analysis of variance (ANOVA) and Tukey’s test (*p* < 0.05) via SPSS version 20.0. Origin software version 9.1 was applied to analyze the data. 

## 3. Results

### 3.1. Physicochemical Properties of Soil and Amendments 

Soil samples had an acidic pH (2.8 ± 0.05) with a soil texture of loamy sand (Table 1). The soil was contaminated with Cu at an ultra-high level of content (9546 ± 5 mg/kg), which far exceeded the Korean standard threshold for farmland soil of 150 mg/kg [28].

**Figure 1:**
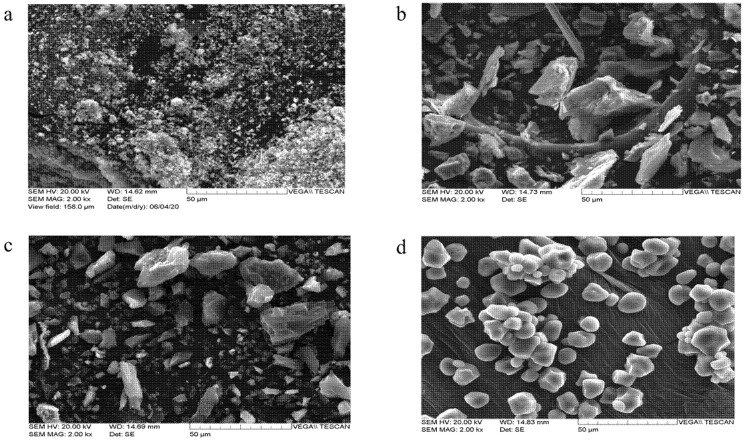
Scanning electron microscopy (SEM) images of (**a**) magnetite, (**b**) talc, (**c**) activated carbon, and (**d**) corn starch.

**Figure 2:**
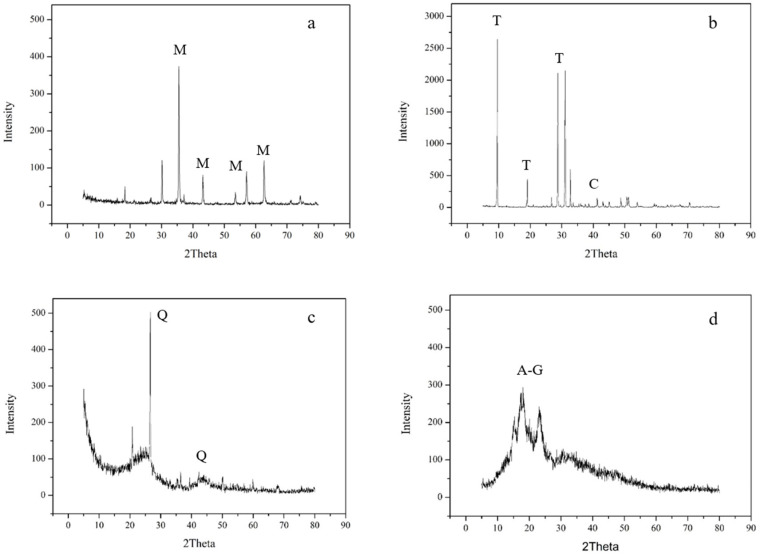
X-ray diffraction (XRD) of (**a**) magnetite, (**b**) talc, (**c**) activated carbon, and (**d**) cornstarch (M: magnetite; T: talc; C: calcite; Q: quartz; A-G: amylose–lipid complex in granules).

In this study, four amendments for high pH and low concentration of Cu were performed, as shown in Table 1. These amendments were characterized by SEM and XRD, as shown in Figure 1 and Figure 2. As shown in Figure 1a, M tended to aggregate and formed inhomogeneous particles [29]. The SEM results for T showed a very heterogeneous particle distribution with an angular structure (Figure 1b). As illustrated in Figure 1c, AC, with a micro-pore structure, was an angular and irregular material [30]. A spherical shape with a homogeneous particle size distribution was found for CS (Figure 1d), which was in good agreement with a previous study [31]. 

The XRD results of the soil amendments are shown in Figure 2. A significant difference in the chemical and structural compositions of amendments was found. The characteristic peaks at diffraction angles 2θ = 35°, 42.5°, 57°, and 62° correspond to the planes (311), (400), (511), and (440) of the M material, which corresponds with magnetite (Figure 2a). These XRD patterns are in good agreement with the Inorganic Crystal Structure Database (ICSD) (Reference Code: 01-076-1849) for magnetite. XRD analysis of T (Figure 2b) showed sharp peaks corresponding to this pure mineral. Diffraction patterns for AC exhibited two prominent broad bands centered around 2θ = 23°, 28°, and 43°. This result indicates the existence of graphite crystallite in AC, which can lead to widening or a disordered internal structure and form a large specific surface area [32]. The XRD diffraction pattern for CS showed distinct peaks in the spectra, demonstrating the crystalline nature of the granules. In addition, a sharp peak at 2θ = 21° suggests the crystalline structure of the amylose–lipid complex in the granules, which is the main component of CS [33].

### 3.2. Effects of Amendments on Cu Removal Rate in the First Stage: Column Leaching Test

**Figure 3:**
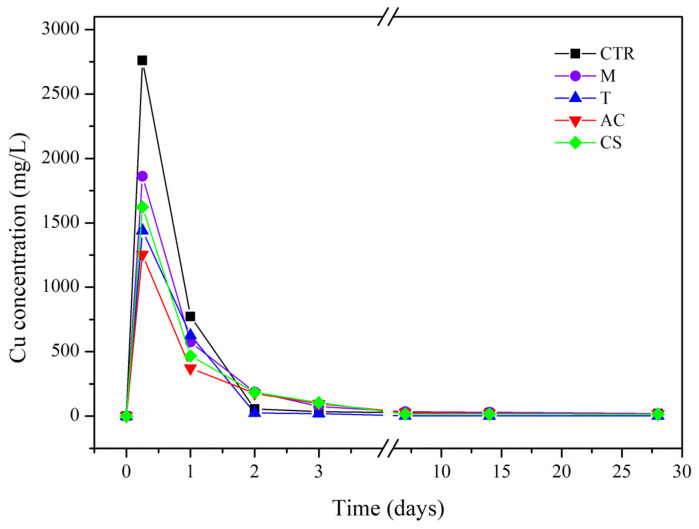
Cu breakthrough curves in column leaching test with and without amendments at a flow rate of 1 mL/min condition (CTR: control; M: magnetite; T: talc; AC: activated carbon; CS: cornstarch).

The purpose of the column leaching test in this study was to evaluate the effect of the applied amendments on Cu immobilization through the loss of Cu in the effluent during the leaching process. The concentration of Cu in the effluent is shown in Figure 3. In all treatments, the concentration of Cu in the effluent rapidly increased after 0.25 days and decreased stably after 3 days. The maximum loss of Cu content was found in the control soil after 0.25 days (2760 mg/L). In the four amendment treatments, the Cu concentration loss was less than the control, and minimal Cu concentration loss was found in the T treatment.

**Figure 4:**
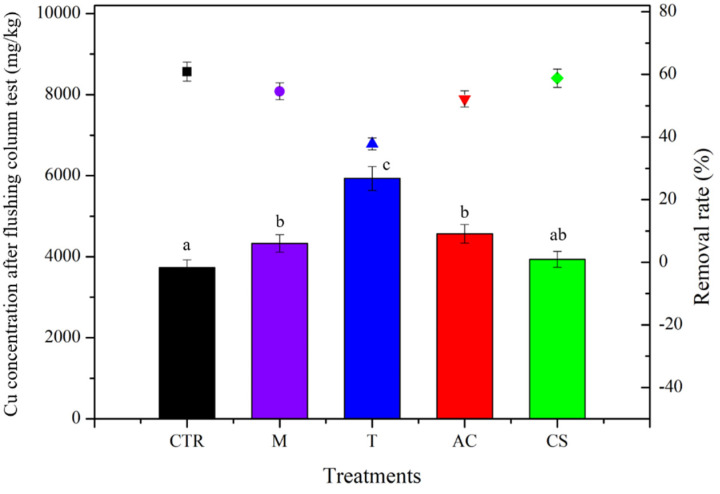
Cu concentration remaining in the soil (bar chart) and the removal rate (symbol dot) after column leaching test with and without amendments at a flow rate of 1 mL/min condition. Means with the same letters are not significantly different from each other under different amendment treatments according to Tukey’s test (*p* < 0.05, *n* = 3) (CTR: control; M: magnetite; T: talc; AC: activated carbon; CS: cornstarch).

After the column leaching test, the concentration of Cu in the soil was measured, as shown in Figure 4. The higher the concentration of Cu that remained in the soil, the lower the removal rate of Cu. As shown in Figure 4, the lowest concentration of Cu remaining in the control soil was 3733 ± 60 mg/kg, as well as the highest removal rate of 60.9%. In contrast, the maximum concentration of Cu was found in the T treatment, which was equivalent to just 37.8% of Cu content removed. These results concur with the result of the Cu breakthrough curve, as shown in Figure 3. 

**Figure 5:**
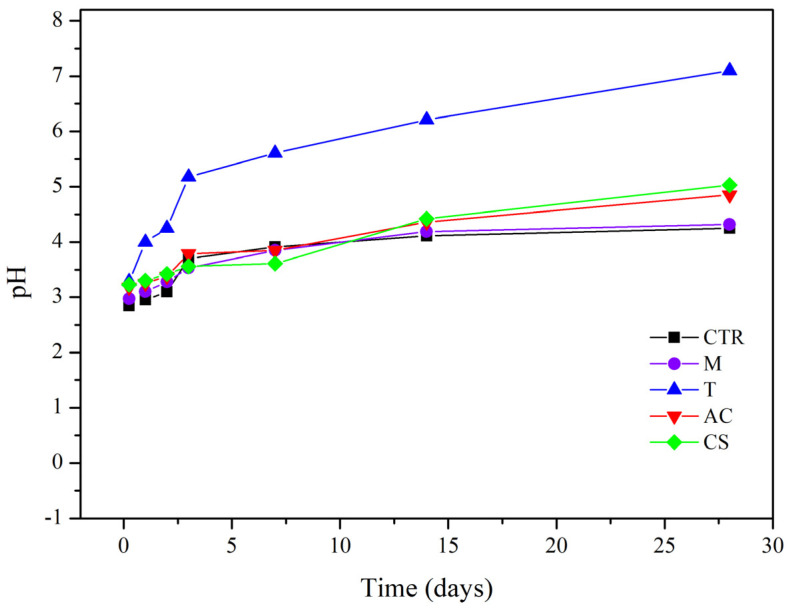
Effluent pH breakthrough curves in column leaching test with and without amendments at a flow rate of 1 mL/min condition (CTR: control; M: magnetite; T: talc; AC: activated carbon; CS: cornstarch).

The concentration of Cu removal in the column leaching test could be related to the pH of the effluent. The effect of amendments on effluent pH in this study is shown in Figure 5. The pH of the effluent increased after 3 days of soil leaching and became stable until 28 days. This study showed that all of the applied amendments changed the effluent pH by increasing the pH from 2.85 in the control soil to 7.1 in the T treatment. The highest efficiency was found in the T treatment by increasing the pH from 3.29 to 7.1 after 28 days.

### 3.3. Effects of Amendments on Cu Removal Rate in the Second Stage: Plant Uptake Test

**Figure 6:**
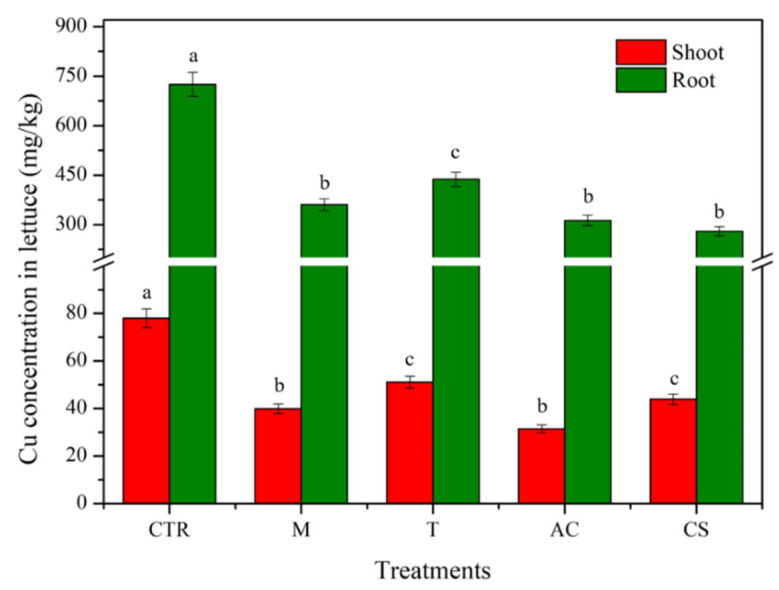
Cu concentration uptake by lettuce in plant uptake test with and without amendments. Means with the same letters are not significantly different from each other under different amendment treatments according to Tukey’s test (*p* < 0.05, *n* = 3) (CTR: control; M: magnetite; T: talc; AC: activated carbon; CS: cornstarch).

The concentration of Cu removal in the second stage (plant uptake test) was described as the Cu concentration uptake by the plant, as shown in Figure 6. The results showed that the soil treated with amendments could reduce the Cu uptake by lettuce compared to the control soil. In detail, amending soil with M, T, AC, and CS significantly (*p* < 0.05) reduced Cu uptake by plant root (360.5 ± 18.0, 437.5 ± 21.8, 312.8 ± 15.6, and 280.1 ± 14.0 mg/kg) and shoot (39.9 ± 1.9, 51.1 ± 2.5, 31.5 ± 1.5, and 43.9 ± 2.2 mg/kg), respectively, compared to the concentration of Cu in lettuce root and shoot of the control soil (725 ± 36.2 and 78 ± 3.9 mg/kg).

**Figure 7:**
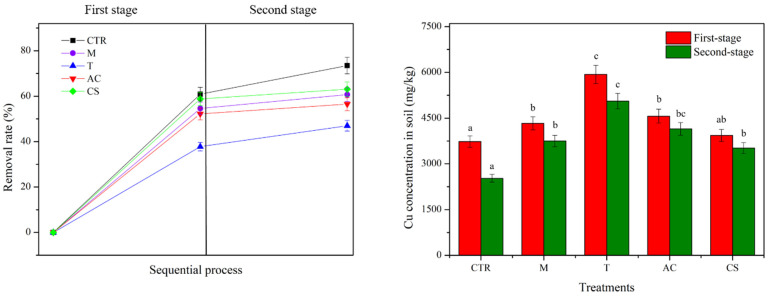
Removal rate of Cu in the soil when assessed by sequential column leaching test and plant uptake test. Means with the same letters are not significantly different from each other under different amendment treatments according to Tukey’s test (*p* < 0.05, *n* = 3) (CTR: control; M: magnetite; T: talc; AC: activated carbon; CS: cornstarch).

The concentration of Cu remaining in the soil when assessed by sequential column leaching and plant uptake tests was used to analyze and calculate the removal rate of Cu after two stages, as summarized in Figure 7. The results showed that after two stages, the Cu removal rate increased from 47% in T to 73.5% in the control soil. The data showed that making amendments to the soil by applying M, T, AC, and CS could immobilize the Cu in the soil, reducing the removal rate of the Cu concentration.

## 4. Discussion

The immobilization technique uses amendments to immobilize heavy metals in soil, thereby rendering them unabsorbable by humans and plants and preventing them from leaching into groundwater [34]. In this study, four amendments, including M, T, AC, and CS, were applied to immobilize the Cu in the ultra-high level of concentration in soil, which meant reducing the Cu mobility in soil. 

The column leaching and plant uptake tests are often used in the removal of heavy-metal-contaminated soil [26,35]. However, this study applied the sequential process of column leaching and plant uptake tests to evaluate the effect of these amendments on Cu immobilization. The importance of applying a sequential process is to provide a method to observe the ability to immobilize Cu in the soil through the percentage of Cu loss (Cu removal rate). 

Generally, all of the amendments used in this study showed promising results in Cu immobilization in the soil with a lower rate of Cu removal compared to the control soil after a sequential process of column leaching and plant uptake tests.

With the presence of the amendments, soil pH was changed, leading to a change in the pH of the leachate. Soil pH is an important factor that can affect the behavior of metals in soil. The results showed that T showed the highest efficiency in increasing soil pH, which changed the pH of the leachate significantly. These results concur with other research, suggesting that T can improve soil pH and further immobilize heavy metals in soil [22,36]. The change in the pH of the leachate in the presence of AC and CS is mainly due to the existence of carbonaceous materials and surface-oxygen-containing functional groups such as carboxyl and hydroxyl groups [37], while the change in pH in the presence of M is attributed to the amount of OH- ions produced in the oxidation process [38]. 

The mechanism of Cu immobilization in soil may be explained by the presence of amendments with useful physicochemical properties. In this study, the immobilization of Cu by M can be attributed to the presence of iron in the amendments, which provides a source of electrons for aqueous reactions and associated contaminant removal [39]. Additionally, the high pH and high specific surface area (45.2 m^2^/g) of M may also contribute to Cu immobilization in the soil through adsorption. Meanwhile, T had the crystals of talc and calcite, which have a chemical composition of Mg_3_Si_4_O_10_(OH)_2_ and CaO, which can increase the pH of soil, resulting in a corresponding increase in the net negative charge of variably charged colloids in sediments such as clays, organic matter, and Fe and Al oxides [40]. This explains why the soil treated with T showed the highest pH of the leachate in the column leaching test and the highest efficiency of Cu immobilization. In addition, Cu is more related to an organic compound, which explains why AC was one of the amendments that affected Cu immobilization in this study. In detail, the results indicated that AC had the highest specific surface area (1082 m^2^/g), which shows its potential capacity for heavy metal adsorption. In addition, AC has a crystal such as quartz, which can induce heavy metal immobilization via cation exchange and surface adsorption. The effect of AC on As, Cu, Zn, and Cd immobilization that was reported in a previous study also concurs with the results of this study [41,42,43]. Finally, CS also had a high pH value (8.2) and the potential capacity for heavy metal immobilization in soil via adsorption, inner-sphere surface complexation, and precipitation techniques. Additionally, many studies have indicated that the functional group in CS such as O-H and C-O also play an important role in Cu, Zn, and Cd adsorption and surface precipitation [44,45].

## 5. Conclusions

This study investigated the effect of various commercial amendments, including magnetite, talc, activated carbon, and cornstarch, on Cu immobilization in soil by considering the loss of Cu during a sequential process of column leaching and plant uptake tests. This study indicated that all applied amendments showed a promising solution for Cu immobilization in soil, where T was found to be the most effective on Cu immobilization. The results showed that the amendments could increase the pH of the soil by increasing the pH of the effluent from 2.85 to 7.1 in the column leaching test. Furthermore, by applying the combination of the sequential process of column leaching and plant uptake tests, the removal rate of Cu was reduced from 73.5% in the control soil to 47.0% with the presence of soil amendments (for T), which indicated that the amendments could reduce Cu mobility and bioavailability and further increase Cu immobilization in soil. This study also aimed to find an effective and promising method that could be used to evaluate Cu immobilization efficiency in soil subjected to remediation on the field scale.

## Figures and Tables

**Table 1:** Physical and chemical properties of the soil and amendments.

Properties	Soil	Amendments			
		M	T	AC	CS
pH	2.8 ± 0.05	8.4 ± 0.04	8.8 ± 0.02	7.2 ± 0.04	8.2 ± 0.01
SSA (m^2^/g)	-	27.37	1.80	1082	0.15
Texture	Loamy sand	-	-	-	-
Cu	9546 ± 5	45.2 ± 0.05	1.4 ± 0.01	4.6 ± 0.03	0.01 ± 0.002

M: magnetite; T: talc; AC: activated carbon; CS: corn starch; SSA: specific surface area.

## Data Availability

The datasets used and/or analyzed during the current study are available from the corresponding author upon reasonable request.

## References

[1] Fajardo C., Ortiz L.T., Rodriguez-Membibre M.L., Nande M., Lobo M.C., Martin M. (2012). Assessing the impact of zero-valent iron (ZVI) nanotechnology on soil microbial structure and functionality: A molecular approach. Chemosphere.

[2] Yan Y., Li Q., Yang J., Zhou S., Wang L., Bolan N. (2020). Evaluation of hydroxyapatite derived from flue gas desulphurization gypsum on simultaneous immobilization of lead and cadmium in contaminated soil. J. Hazard. Mater..

[3] Wang P., Chen H., Kopittke P.M., Zhao F.-J. (2019). Cadmium contamination in agricultural soils of China and the impact on food safety. Environ. Pollut..

[4] Boudebbouz A., Boudalia S., Bousbia A., Habila S., Boussadia M.I., Gueroui Y. (2021). Heavy metals levels in raw cow milk and health risk assessment across the globe: A systematic review. Sci. Total Environ..

[5] Cui H., Bao B., Cao Y., Zhang S., Shi J., Zhou J., Zhou J. (2022). Combined application of ferrihydrite and hydroxyapatite to immobilize soil copper, cadmium, and phosphate under flooding-drainage alternations. Environ. Pollut..

[6] Rizwan M.S., Imtiaz M., Zhu J., Yousaf B., Hussain M., Ali L., Ditta A., Zahid Ihsan M., Huang G., Ashraf M. (2021). Immobilization of Pb and Cu by organic and inorganic amendments in contaminated soil. Geoderma.

[7] Li C., Zhou K., Qin W., Tian C., Qi M., Yan X., Han W. (2019). A Review on Heavy Metals Contamination in Soil: Effects, Sources, and Remediation Techniques. Soil Sediment Contam. Int. J..

[8] Yao Z., Li J., Xie H., Yu C. (2012). Review on Remediation Technologies of Soil Contaminated by Heavy Metals. Procedia Environ. Sci..

[9] Mahar A., Wang P., Li R., Zhang Z. (2015). Immobilization of Lead and Cadmium in Contaminated Soil Using Amendments: A Review. Pedosphere.

[10] Nguyen Quoc T., Kim J.W., Nejad Z.D., Le Thanh T., Jung M.C. (2022). Influence of commercial amendments on Cu and Zn mobility, phytoavailability, and microbial activities on two contaminated soils. J. Environ. Chem. Eng..

[11] Zaitan H., Bianchi D., Achak O., Chafik T. (2008). A comparative study of the adsorption and desorption of o-xylene onto bentonite clay and alumina. J. Hazard. Mater..

[12] Gilmour C.C., Riedel G.S., Riedel G., Kwon S., Landis R., Brown S.S., Menzie C.A., Ghosh U. (2013). Activated carbon mitigates mercury and methylmercury bioavailability in contaminated sediments. Environ. Sci. Technol..

[13] Meynet P., Hale S.E., Davenport R.J., Cornelissen G., Breedveld G.D., Werner D. (2012). Effect of activated carbon amendment on bacterial community structure and functions in a PAH impacted urban soil. Environ. Sci. Technol..

[14] Xie Y., Xiao K., Sun Y., Gao Y., Yang H., Xu H. (2018). Effects of amendments on heavy metal immobilization and uptake by Rhizoma chuanxiong on copper and cadmium contaminated soil. R. Soc. Open Sci..

[15] Gomez-Eyles J.L., Yupanqui C., Beckingham B., Riedel G., Gilmour C., Ghosh U. (2013). Evaluation of biochars and activated carbons for in situ remediation of sediments impacted with organics, mercury, and methylmercury. Environ. Sci. Technol..

[16] Wu B., Cheng G., Jiao K., Shi W., Wang C., Xu H. (2016). Mycoextraction by Clitocybe maxima combined with metal immobilization by biochar and activated carbon in an aged soil. Sci. Total Environ..

[17] León O., Soto D., González J., Piña C., Muñoz-Bonilla A., Fernandez-García M. (2019). Environmentally Friendly Fertilizers Based on Starch Superabsorbents. Materials.

[18] Baragaño D., Alonso J., Gallego J.R., Lobo M.C., Gil-Díaz M. (2020). Magnetite nanoparticles for the remediation of soils co-contaminated with As and PAHs. Chem. Eng. J..

[19] Huang R., Li Y., Li F., Yin X., Li R., Wu Z., Liang X., Li Z. (2022). Phosphate fertilizers facilitated the Cd contaminated soil remediation by sepiolite: Cd mobilization, plant toxicity, and soil microbial community. Ecotoxicol. Environ. Saf..

[20] Sabir M., Hanafi M.M., Aziz T., Ahmad H.R., Zia-Ur-Rehman M., Saifullah U., Murtaza G., Hakeem K.R. (2013). Comparative effect of activated carbon, pressmud and poultry manure on immobilization and concentration of metals in maize (*Zea mays*) grown on contaminated soil. Int. J. Agric. Biol..

[21] Zhang W.-H., Sun R.-B., Xu L., Liang J.-N., Zhou J. (2019). Assessment of bacterial communities in Cu-contaminated soil immobilized by a one-time application of micro-/nano-hydroxyapatite and phytoremediation for 3 years. Chemosphere.

[22] Nguyen Quoc T., Nejad Z.D., Jung M.C. (2021). Effect of Commercial Amendments on Immobilization of Arsenic, Copper, and Zinc in Contaminated Soil: Comprehensive Assessing to Plant Uptake Combined with a Microbial Community Approach. Minerals.

[23] Bakircioglu D., Kurtulus Y.B., İbar H. (2011). Comparison of extraction procedures for assessing soil metal bioavailability of to wheat grains. Clean–Soil Air Water.

[24] Sims J., Johnson G. (1991). Micronutrient soil tests. Micronutr. Agric..

[25] Gee G., Bauder J. (1986). Particle-Size Analysis 1. Methods of Soil Analysis: Part 1—Physical and Mineralogical Methods. Agron. Monogr..

[26] Chen G.-C., Liu Z., Zhang J., Owens G. (2012). Phytoaccumulation of copper in willow seedlings under different hydrological regimes. Ecol. Eng..

[27] Derakhshan Nejad Z., Rezania S., Jung M.C., Al-Ghamdi A.A., Mustafa A.E.-Z.M.A., Elshikh M.S. (2021). Effects of fine fractions of soil organic, semi-organic, and inorganic amendments on the mitigation of heavy metal(loid)s leaching and bioavailability in a post-mining area. Chemosphere.

[28] KMOE (2013). Detailed Survey for Soil and Water Contamination in Abandoned Metal Mines in Korea.

[29] del Campo A., Sen T., Lellouche J.-P., Bruce I.J. (2005). Multifunctional magnetite and silica–magnetite nanoparticles: Synthesis, surface activation and applications in life sciences. J. Magn. Magn. Mater..

[30] Benedetti V., Patuzzi F., Baratieri M. (2018). Characterization of char from biomass gasification and its similarities with activated carbon in adsorption applications. Appl. Energy.

[31] Ciesielskia W., Lii C.-y., Yen M.-T., Tomasik P. (2003). Interactions of starch with salts of metals from the transition groups. Carbohydr. Polym..

[32] Xie Z., Guan W., Ji F., Song Z., Zhao Y. (2014). Production of Biologically Activated Carbon from Orange Peel and Landfill Leachate Subsequent Treatment Technology. J. Chem..

[33] Arık Kibar A., Gönenç İ., Us F. (2010). Gelatinization of waxy, normal and high amylose corn starches. GIDA.

[34] Bolan N.S., Duraisamy V. (2003). Role of inorganic and organic soil amendments on immobilisation and phytoavailability of heavy metals: A review involving specific case studies. Soil Res..

[35] Wang G., Zhang S., Zhong Q., Xu X., Li T., Jia Y., Zhang Y., Peijnenburg W.J., Vijver M.G. (2018). Effect of soil washing with biodegradable chelators on the toxicity of residual metals and soil biological properties. Sci. Total Environ..

[36] Warton B., Matthiessen J.N. (2005). The crucial role of calcium interacting with soil pH in enhanced biodegradation of metam-sodium. Pest Manag. Sci..

[37] Zhu J., Gao W., Ge L., Zhao W., Zhang G., Niu Y. (2021). Immobilization properties and adsorption mechanism of nickel(II) in soil by biochar combined with humic acid-wood vinegar. Ecotoxicol. Environ. Saf..

[38] Danila V., Kumpiene J., Kasiuliene A., Vasarevičius S. (2020). Immobilisation of metal(loid)s in two contaminated soils using micro and nano zerovalent iron particles: Evaluating the long-term stability. Chemosphere.

[39] Fajardo C., Costa G., Nande M., Martín C., Martín M., Sánchez-Fortún S. (2019). Heavy metals immobilization capability of two iron-based nanoparticles (nZVI and Fe_3_O_4_): Soil and freshwater bioassays to assess ecotoxicological impact. Sci. Total Environ..

[40] Zhang Y., Zhang H., Wang M., Zhang Z., Marhaba T., Sun C., Zhang W. (2019). In situ immobilization of heavy metals in contaminated sediments by composite additives of hydroxyapatite and oxides. Environ. Earth Sci..

[41] de Oliveira L.M., Suchismita D., Gress J., Rathinasabapathi B., Chen Y., Ma L.Q. (2017). Arsenic uptake by lettuce from As-contaminated soil remediated with *Pteris vittata* and organic amendment. Chemosphere.

[42] Que W., Zhou Y.-h., Liu Y.-g., Wen J., Tan X.-f., Liu S.-j., Jiang L.-h. (2019). Appraising the effect of in-situ remediation of heavy metal contaminated sediment by biochar and activated carbon on Cu immobilization and microbial community. Ecol. Eng..

[43] Brendova K., Zemanova V., Pavlikova D., Tlustos P. (2016). Utilization of biochar and activated carbon to reduce Cd, Pb and Zn phytoavailability and phytotoxicity for plants. J. Environ. Manag..

[44] Pourbeyram S. (2016). Effective Removal of Heavy Metals from Aqueous Solutions by Graphene Oxide-Zirconium Phosphate (GO-Zr-P) Nanocomposite. Ind. Eng. Chem. Res..

[45] Huang Q., Chen Y., Yu H., Yan L., Zhang J., Wang B., Du B., Xing L. (2018). Magnetic graphene oxide/MgAl-layered double hydroxide nanocomposite: One-pot solvothermal synthesis, adsorption performance and mechanisms for Pb^2+^, Cd^2+^, and Cu^2+^. Chem. Eng. J..

